# Mapping visual symbols onto spoken language along the ventral visual stream

**DOI:** 10.1073/pnas.1818575116

**Published:** 2019-08-19

**Authors:** J. S. H. Taylor, Matthew H. Davis, Kathleen Rastle

**Affiliations:** ^a^School of Life and Health Sciences, Aston University, Birmingham B4 7ET, United Kingdom;; ^b^Medical Research Council Cognition and Brain Sciences Unit, University of Cambridge, Cambridge CB2 7EF, United Kingdom;; ^c^Department of Psychology, Royal Holloway University of London, Egham TW20 0EX, United Kingdom

**Keywords:** orthography, fMRI, representation, learning, reading

## Abstract

Reading involves transforming arbitrary visual symbols into sounds and meanings. This study interrogated the neural representations in ventral occipitotemporal cortex (vOT) that support this transformation process. Twenty-four adults learned to read 2 sets of 24 novel words that shared phonemes and semantic categories but were written in different artificial orthographies. Following 2 wk of training, participants read the trained words while neural activity was measured with functional MRI. Representational similarity analysis on item pairs from the same orthography revealed that right vOT and posterior regions of left vOT were sensitive to basic visual similarity. Left vOT encoded letter identity and representations became more invariant to position along a posterior-to-anterior hierarchy. Item pairs that shared sounds or meanings, but were written in different orthographies with no letters in common, evoked similar neural patterns in anterior left vOT. These results reveal a hierarchical, posterior-to-anterior gradient in vOT, in which representations of letters become increasingly invariant to position and are transformed to convey spoken language information.

## Significance

Learning to read is the most important milestone in a child’s education. However, controversies remain regarding how readers’ brains transform written words into sounds and meanings. We address these by combining artificial language learning with neuroimaging to reveal how the brain represents written words. Participants learned to read new words written in 2 different alphabets. Following 2 wk of training, we found a hierarchy of brain areas that support reading. Letter position is represented more flexibly from lower to higher visual regions. Furthermore, higher visual regions encode information about word sounds and meanings. These findings advance our understanding of how the brain comprehends language from arbitrary visual symbols.

Reading acquisition requires the brain to abstract away from the visual forms of written words to access spoken language information. This abstraction requires encoding distinctive information about each visual symbol (e.g., “d” has a circle to the left, and “b” has a circle to the right), but in a way that permits recognition irrespective of variations in case, font, size (1, 2), or position in a word (e.g., the b in Cab is the same as the B in Bad) (3). For skilled readers, this process culminates in an inextricable link between the perception of a word’s visual form and the stored linguistic knowledge it represents (4). The current study delineates how representations along the ventral visual stream support this transformation.

Neuroimaging research suggests that abstraction away from veridical visual form in reading is achieved by left ventral occipitotemporal cortex (vOT). Neural priming effects are observed in this region for cross-case (e.g., rage−RAGE) and location-shifted (e.g., #RAGE−RAGE#) written word pairs (5, 6). Patterns of activity across voxels in left vOT are also more similar for pairs of letters with the same abstract identity (e.g., R and r) than for letter pairs sharing visual, phonological, or motoric features (7). Dehaene et al. (8) proposed that, from posterior-to-anterior left vOT, neural representations become increasingly invariant to retinal location and encode increasingly complex orthographic information. Supporting this, along this axis, left vOT shows a gradient of selectivity for the word likeness of written forms (9). Representations in middle-to-anterior left vOT also appear to be sensitive to higher-level language information (10–12). For example, this region shows masked neural priming effects for word−picture pairs that have the same spoken form and represent the same concept (e.g., a picture of a lion primed the word LION, and vice versa; ref. 13). However, while existing research implicates the left vOT in encoding important information during reading, the nature of the representations that support this process are not well specified.

**Fig. 1.**
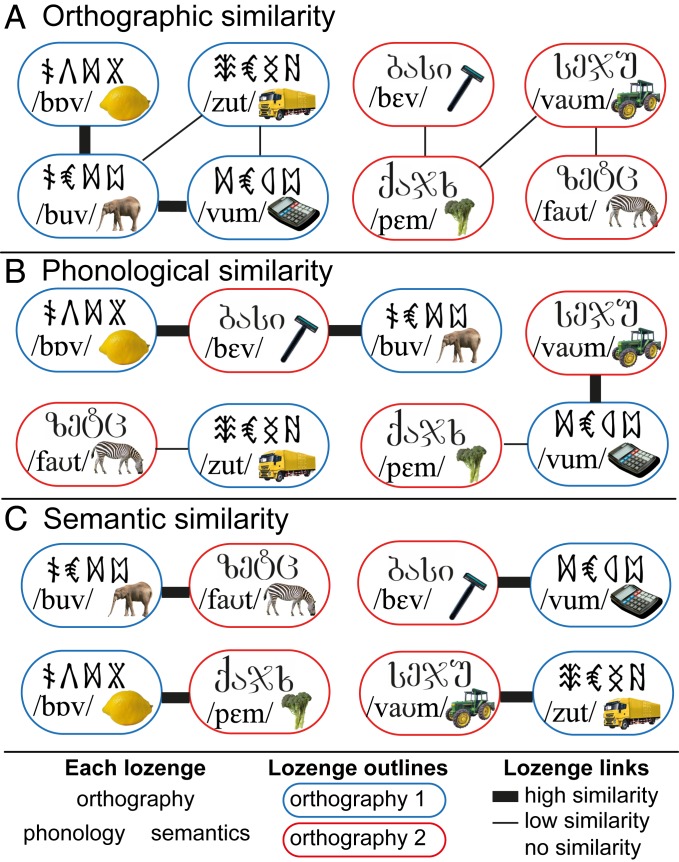
Schematic of 3 possible similarity structures between a subset of the trained words. Each lozenge contains the orthographic, phonological, and semantic form of an item, with items from one orthography in blue lozenges, and those from the other in red lozenges. A thicker line between pairs indicates greater similarity. (*A*) Orthographic similarity reflects the number of symbols (out of 4) shared in the same position, although analyses also examined symbols shared across positions. (*B*) Phonological similarity reflects the number of phonemes (out of 3) shared in the same position. (*C*) Semantic similarity reflects shared semantic category. Note that phonological and semantic similarity analyses excluded within-orthography pairs, and so were not confounded by orthographic similarity.

**Fig. 2.**
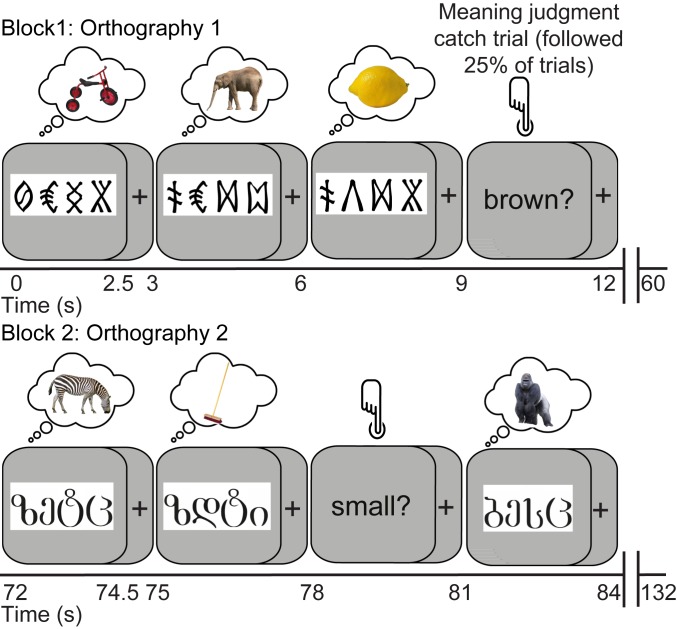
The fMRI scanning procedures. Participants viewed and covertly retrieved the meanings of the trained words while neural activity was measured with fMRI (acquisition/repetition time 2 s). The 2 orthographies were presented in alternating 60-s blocks, and each item (*n* = 24 per orthography) was presented 16 times across four 15-min runs.

**Fig. 3.**
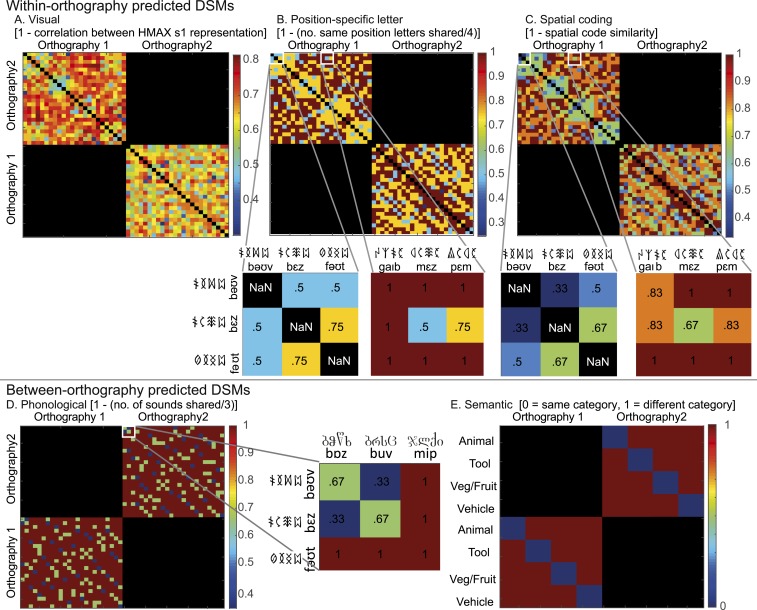
Predicted dissimilarity matrices for the learned words based on (*A*) 1 minus correlation between s1 layer representations from the HMAX model, (*B*) 1 minus proportion of shared same-position letters, (*C*) 1 minus spatial coding similarity, in which the similarity between item pairs is graded according to the distance in position between shared letters, (*D*) 1 minus proportion of shared same-position phonemes, and (*E*) shared (0) or not shared (1) semantic category. *A*–*C* included only within-orthography pairs, since items written in different orthographies share no letters. *D* and *E* included only between-orthography pairs to ensure that effects were specific to shared sounds or meanings, not shared letters. Note that the assignment of orthography to phonological forms and meanings was counterbalanced across participants; therefore, the visual and semantic predicted DSMs shown are those used for half of the participants (*SI Appendix*, *SI Methods*). The Spearman correlations among the within-orthography DSMs are visual and position-specific letter DSMs (*r* = 0.53, *r* = 0.51, for each half of the participants), visual and spatial coding DSMs (*r* = 0.48, *r* = 0.43), and position-specific letter and spatial coding DSMs (*r* = 0.86).

The current study used representational similarity analysis (RSA) of brain responses measured with functional MRI (fMRI) to delineate how the vOT processing stream encodes information about written words to support computation of higher-level language information. In particular, we sought to uncover how vOT represents letter identity and position, and the extent to which representations along this pathway come to capture word sounds and meanings. To do so, we trained participants for 2 wk to read 2 sets of pseudowords constructed from 2 different artificial orthographies. Each item had a distinct meaning and comprised 4 symbols, 3 representing the pseudoword phonemes and a final silent symbol. Phonemes and semantic categories were shared between the 2 orthographies and, for each participant, one orthography had a systematic mapping between the final symbol of each word and the word’s semantic category (see *SI Appendix*, *SI Methods* for details). This allowed us to manipulate word form, sound, and meaning (Fig. 1) in a manner that would be hard to achieve in natural languages (however, see refs. 12 and 14). Following training, we examined the multivoxel patterns of fMRI responses (for an illustration of this method, see ref. 7) evoked when participants covertly retrieved the meanings of the newly learned written words (see Fig. 2 for scanning paradigm). Our analyses (see Fig. 3 for predicted models of similarity) sought to determine whether and how representations in vOT capture the separate orthographic, phonological, and semantic similarity across newly learned words.

## Results

### Reading Artificial Orthographies Evokes Extensive Activity in vOT (Fig. 4).

Following 9 d of training on the new words written in the 2 orthographies, 24 native English-speaking adults gave correct meanings for 92% (SD = 16%) of items and correct pronunciations for 86% (SD = 22%) of items (all results collapsed across the 2 orthographies; no significant differences between them). They also pronounced 79% (SD = 33%) of untrained items correctly, indicating extraction of individual symbol–sound mappings (see also ref. 15). Furthermore, trained items (mean response time 1,731 ms, SD = 592 ms) were read faster than untrained items (2,716 ms, SD = 988 ms, *t*[22] = 6.05, *P* < 0.001), mirroring reading speeds for words versus pseudowords in familiar orthographies such as English, and replicating previous work using artificial orthographies (16, 17).

**Fig. 4.**
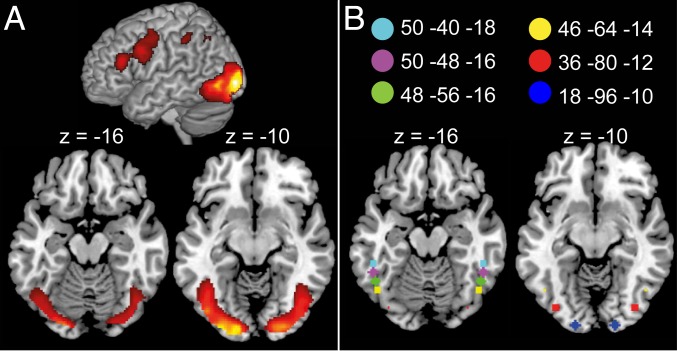
(*A*) Univariate activation during reading of trained words, *P* < 0.001 uncorrected, *P* < 0.05 familywise error cluster extent corrected. (*B*) Location of 4-mm-radius spherical vOT ROIs taken from ref. 9.

Fig. 4*A* (*SI Appendix*, Table S1) shows significant activation of bilateral occipitotemporal cortices, bilateral inferior and superior parietal cortices, left inferior frontal and precentral gyri, and the supplementary motor area, when participants covertly read the learned words. Activation was assessed relative to the unmodeled resting baseline, excluding meaning judgment catch trials, during scanning runs that took place after the last day of training. As in previous work with artificial orthographies (17), these regions closely correspond to those activated when adults read words written in natural alphabetic languages (18). Subsequent analyses focus on six 4-mm-radius spherical regions of interest (ROIs) in bilateral vOT (Fig. 4*B*). From posterior to anterior, the ROIs were located in inferior occipital cortex/lingual gyrus (ROI 1), fusiform gyrus/inferior occipital cortex (ROI 2), inferior occipital cortex (ROI 3), and inferior temporal gyrus (ROIs 4 to 6) (anatomical labels were generated by MRICron; ref. 19, based on ref. 20). These ROIs were selected a priori from published literature showing increasingly selective responses to word-like stimuli from posterior-to-anterior vOT (9).

### Posterior and Right vOT Neural Response Patterns Are Sensitive to Basic Visual Similarity (Fig. 5).

**Fig. 5.**
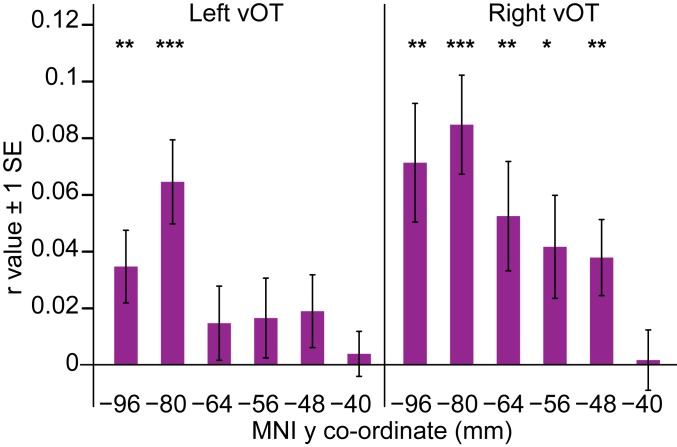
Correlations between the neural and visual DSM in left- and right-hemisphere ROIs, from posterior to anterior vOT along the *x* axis. MNI y coordinates express the distance in millimeters from the anterior commissure to the center of each ROI in Fig. 4*B*. Asterisks denote whether second-level one-sample *t* tests in each ROI indicated a significantly greater than zero correlation (one-tailed *t* test, ***P < 0.001, **P < 0.01, *P < 0.05). SE bars are appropriate for these one-sample *t* tests.

We first determined whether vOT representations of newly learned words are sensitive to their low-level visual similarity. We constructed a visual dissimilarity matrix (predicted DSM) using the simple cell representations (s1 layer) from the Hierarchical Model and X (HMAX) model of visual object recognition, which comprises Gabor filters of varying orientation and size (ref. 21; see also ref. 22). This visual DSM was computed as 1 minus the Pearson correlation between the s1 layer representations for all word pairs from within the same orthography (Fig. 3*A*). We computed a neural DSM, the voxel-wise dissimilarity (1 minus the Pearson correlation) between responses to all within-orthography word pairs in searchlights across the whole brain. We then conducted a Spearman correlation between the predicted DSM and neural DSM (see *SI Appendix*, Fig. S1 and Table S2 for whole-brain results). The mean correlations for each participant were extracted from vOT ROIs using MarsBaR (see also ref. 23) and submitted to second-level one-sample *t* tests to identify ROIs in which the correlation was greater than zero. The visual DSM was positively correlated with the neural response patterns in all right-hemisphere vOT ROIs except the most anterior, but only in the 2 most posterior left-hemisphere vOT ROIs. Fig. 5 and *SI Appendix*, Table S5 show this posterior and right-hemisphere distribution of sensitivity to basic visual form, which was confirmed with an ANOVA that obtained main effects of hemisphere, *F*(1,23) = 5.12, *P* = 0.03, *η*^2^ = 0.02, and region, *F*(3.50, 80.54) = 8.17, *P* < 0.001, *η*^2^ = 0.09, with no interaction between them, *F*(3.17, 72.84) < 1 (Greenhouse Geisser correction applied where Mauchly’s test indicated that the assumption of sphericity was violated).

### Letter Representations Are More Invariant across Position in Anterior than Posterior vOT (Fig. 6).

We computed a position-specific letter DSM (Fig. 3*B*) as 1 minus the proportion of same-position letters shared between all within-orthography word pairs, and a more position-invariant letter DSM (Fig. 3*C*), in which the similarity between items pairs is graded according to the distance in position between shared letters (spatial coding model; ref. 3). These were both correlated with the neural response patterns in all 6 ROIs in left and right vOT (*SI Appendix*, Fig. S1 and Tables S3–S5). While results from the visual DSM suggest that representations in right and posterior left vOT reflect aspects of visual form, these analyses suggest that left midanterior vOT represents a word’s component letters.

**Fig. 6.**
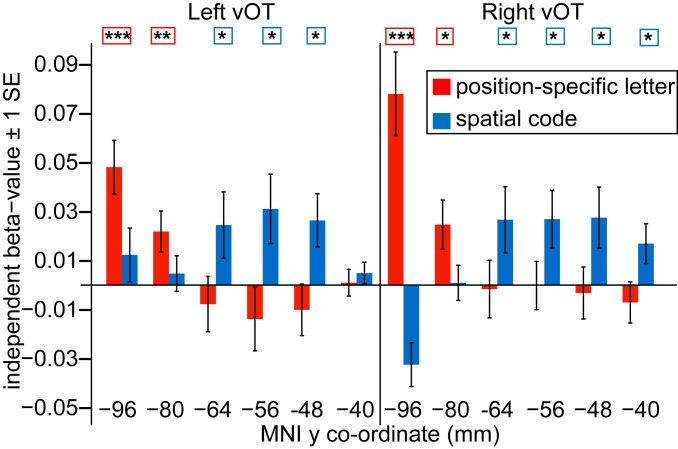
Results of a simultaneous multiple regression analysis examining the independent variance in the neural DSM accounted for by the position-specific letter and spatial coding DSMs. Left- and right-hemisphere ROIs go from posterior to anterior vOT along the *x* axis. Red and blue bars show the mean independent beta value for the position-specific letter and spatial coding DSMs. Asterisks denote whether second-level one-sample *t* tests on the resulting beta values for each predicted DSM in each ROI were significantly greater than zero (one-tailed *t* test, ***P < 0.001, **P < 0.01, *P < 0.05). SE bars are appropriate for these one-sample *t* tests.

We next investigated whether letter representations become more invariant to position along the vOT processing hierarchy. We conducted a multiple regression analysis (see *SI Appendix*, *SI Results* for justification) in searchlights across the whole brain, including both the position-specific and spatial coding DSMs as predictors, and extracted the independent beta values for each model from the vOT ROIs. As shown in Fig. 6, this analysis revealed that, in both left and right vOT, the position-specific letter DSM accounted for significant independent variance in the neural response patterns in the 2 most posterior ROIs, whereas the spatial coding DSM accounted for significant independent variance in the middle-to-anterior vOT ROIs. An ANOVA on the beta values confirmed that the variance accounted for by the 2 DSMs differed across these ROIs (region × DSM interaction, *F*[2.80,64.40] = 13.60, *P* < 0.001, *η*^2^ = 0.13; no 3-way interaction with hemisphere, *F*[2.74,63.07] = 2.26, *P* = 0.096). These results suggest that the representation of letter identity is tied to information about letter position in bilateral posterior vOT but not in bilateral midanterior vOT, and that spatial coding provides one candidate model for characterizing how the position of letters within words is represented in these more anterior vOT regions (see *SI Appendix*, *SI Results* for an alternative open-bigram coding model).

### Middle-to-Anterior vOT Response Patterns Capture Phonological and Semantic Similarity (Fig. 7).

**Fig. 7.**
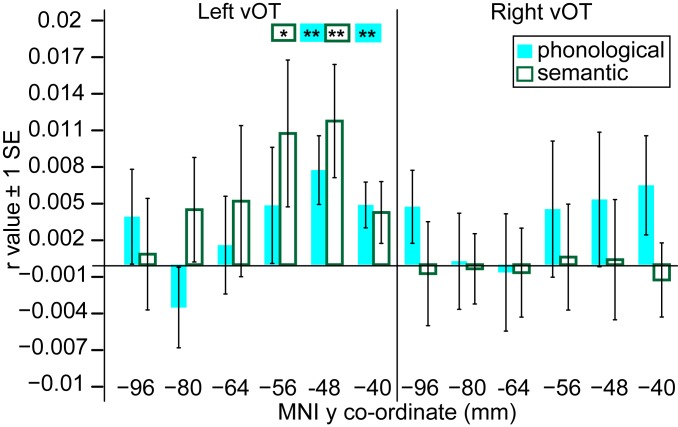
Correlations between the neural and phonological (cyan) and semantic (green) DSMs in left- and right-hemisphere ROIs, from posterior to anterior vOT along the *x* axis. Asterisks denote whether second-level one-sample *t* tests in each ROI indicated a significantly greater than zero correlation (one-tailed *t* test, **P < 0.01, *P < 0.05). SE bars are appropriate for these one-sample *t* tests.

Our final analyses examined whether the vOT processing hierarchy encodes the phonological and semantic properties of written words. We used a phonological predicted DSM, computed as 1 minus the proportion of same-position phonemes shared between item pairs (Fig. 3*D*). Crucially, this included only between-orthography pairs, such that similarity was based on shared phonemes for items that shared no letters. Second-level one-sample *t* tests demonstrated a significant correlation between the phonological DSM and neural response patterns in the 2 most anterior left vOT ROIs, but not with those in more posterior left vOT ROIs or right-hemisphere ROIs (Fig. 7, whole-brain searchlight results in *SI Appendix*, Fig. S3 and Table S7). Thus, neural representations in left inferior temporal gyrus reflect information about phonological form, independent of orthographic form.

Neural representations in middle-to-anterior left vOT were also sensitive to the semantic similarity between items. We computed a semantic category predicted DSM, in which items from different semantic categories were classed as dissimilar and those from the same category as similar (Fig. 3*E*). Again, only between-orthography pairs were included to ensure that results were driven by semantic and not orthographic similarity. The semantic category DSM was positively correlated with the neural response patterns in the second 2 most anterior left vOT ROIs, but not with those in more posterior left vOT ROIs or right-hemisphere ROIs (Fig. 7, whole-brain searchlight results in *SI Appendix*, Fig. S3 and Table S8). Thus, left inferior temporal gyrus also encoded semantic information about the newly learned words.

## Discussion

Reading requires the brain to map visual information onto language information. Research suggests that the ventral visual stream plays a key role in this process, but the nature of the representations that underpin this transformation remain unspecified. Using RSA, we demonstrated that, whereas right vOT and left posterior inferior occipital cortex represent written words in terms of their low-level visual form, left middle-to-anterior vOT represents words in terms of their letters. Furthermore, these orthographic representations become progressively more abstract along the posterior-to-anterior processing hierarchy. The response patterns indicated that information about letter identity is more invariant across position in anterior inferior occipital cortex and inferior temporal gyrus than in posterior inferior occipital cortex. Transformation away from veridical visual form was even greater in midanterior left inferior temporal gyrus, where representations were sensitive to shared sounds and semantic categories of words written in different orthographies. Our research thus demonstrates how the ventral stream transforms visual inputs to meaningful linguistic information, and reveals the representations that make this possible.

Substantial research in cognitive psychology has sought to specify the nature of the orthographic codes that support visual word recognition (24). Various computational models consider the challenge of mapping retinotopically organized visual information onto location-invariant orthographic representations that specify within-word letter position, for example using spatial (3) or open bigram (24, 25) coding. Our results provide support for these cognitive models. Neural patterns in middle-to-anterior vOT were better characterized by spatial coding than by position-specific letter coding, and a supplementary analysis also showed this to be true for open-bigram relative to position-specific coding. However, while position-specific letter coding characterized response patterns in posterior vOT better than spatial (or open-bigram) coding, neural responses in these regions could also be accounted for by a visual model (21). These data therefore support cognitive models in suggesting that location specificity gives way to more location-invariant representations at hierarchically higher levels of the ventral processing stream (*SI Appendix*, *SI Discussion*). However, further work is necessary to determine exactly how this location invariance is achieved (whether by multiletter representations or something more akin to spatial coding) and, more broadly, to examine whether and where vOT representations of written words are better characterized by these visual word recognition models than by generic models of visual processing, such as HMAX (21, 26).

Location-invariant letter coding (a hallmark of skilled reading; ref. 25) is critical for establishing the mapping between written and spoken language because it allows each experience of a letter to converge on the same spoken language representation. The observation that neural response patterns were similar for words that shared sounds, despite sharing no letters, indicates that phonological information shapes representations in midanterior regions of the left vOT hierarchy. One interpretation of this result is that representations in this part of the left vOT processing pathway are phonological. However, an intriguing alternative is that this result reflects the emergence of abstract letter identities (ALIs) for symbols from the 2 orthographies that correspond to the same sound. The existence of such ALIs is supported by studies of cross-case (e.g., rage−RAGE) and cross-script (e.g., Japanese Kanji−Kana) similarity using behavioral (1, 2, 27) and neural (5–7, 28) measures (although the orthographic nature of cross-script effects is still debated; refs. 29). By this account, phonology is not represented in left vOT, but nonetheless plays a crucial role in shaping the abstract orthographic representations in this region, since it is shared sounds that bind together these cross-script visual forms.

An overlapping region of left midanterior vOT encoded information about the meanings of the words, showing similar neural response patterns for item pairs that were from the same semantic category, but shared no letters. This finding is in line with previous neuroimaging studies (11, 12) and with the view that the ventral reading pathway maps from word form to meaning (18). By examining the full vOT hierarchy and disentangling orthographic, phonological, and semantic similarity, we have shown that, even with relatively little experience of a writing system, left midanterior vOT representations capture the spoken language associations of written words. Further research should establish whether phonological and semantic information is intrinsically represented in this region, or whether these aspects of spoken language shape organization of orthographic representations through interactions with other brain areas (30). It will also be important to specify how linguistic influences on vOT change over time; both in the short term while reading a word (10, 13) and during the long process of reading development (31).

In summary, our study provides strong empirical support for a hierarchical, posterior-to-anterior gradient in vOT that represents increasingly abstract information about written words. In line with Dehaene et al.’s (8) proposal, we found that representations in posterior visual regions are tied to location and may encode low-level visual information, whereas letter identity was represented in left midanterior vOT with a degree of location invariance. These location-invariant letter representations are then further transformed in left midanterior vOT to encode aspects of a word’s pronunciation and meaning. These results contribute to our understanding of how the brain maps from arbitrary visual symbols to rich linguistic representations, ultimately enabling the experience of language through the visual modality.

## Methods

Materials and datasets are available at refs. 32 and 33.

### Participants.

Twenty-four native English-speaking students (19 females) aged 18 to 30 y from Royal Holloway University of London (RHUL) participated. Participants were right-handed with no history of learning disabilities or hearing or vision impairments. RHUL Ethics Committee approved the research. Participants signed an informed consent form, and were paid for participation.

### Stimuli and Behavioral Training.

Over 9 sessions, participants learned to read 2 sets of 24 consonant−vowel−consonant pseudowords written in 2 different unfamiliar alphabets and assigned an English common noun meaning. Each item comprised 4 symbols; 3 corresponding to the pseudoword phonemes, and a final silent symbol. Training tasks required mapping between written form and sound or meaning (see *SI Appendix*, *SI Methods* for details).

### MRI Scanning Procedure.

After behavioral training, participants completed eight 15-min fMRI scanning runs over 2 d. Runs alternated between visual (orthographic forms) and auditory (phonological forms) presentation of the words. Only visual runs are reported here (paradigm shown in Fig. 2). Visual stimuli were projected onto a screen at the rear end of the scanner bore and viewed via a mirror mounted on the head coil. Each stimulus was 320 × 112 pixels presented at a distance of 77 cm giving an image of 6.30° × 2.20° visual angle. In each visual run, the orthographic form of each trained item was presented 4 times, with the order of item presentation and repetition randomized within run. On each trial, participants were instructed to think about the item’s meaning. Catch trials followed 25% of trials (one per item) and presented a single word question on the screen (small?, dangerous?, heavy?, long?, Britain?). Participants used a button box to respond YES or NO with respect to the previous item. Performance was 84% correct (SD = 13%). Trials were 2,500 ms, with a 500-ms intertrial interval. Runs were split into 20 trial blocks (16 standard, 4 catch), alternating between the 2 orthographies. Blocks were separated by a 12-s rest period (blank screen). After the first 5 participants, we decided to monitor attention during scanning. Therefore, for participant 6 onward, after blocks 1, 4, 7, and 10, participants saw a feedback screen that read “100% - well done!”, or “25/50/75% - oops try and concentrate!,” indicating the percentage of catch trials on which they had responded. Functional imaging acquisition parameters and preprocessing details are given in *SI Appendix*, *SI Methods*.

### fMRI Analyses.

Smoothed, normalized functional images were used for univariate analyses, whereas, for multivariate analyses, we used unsmoothed native-space images. For both analyses, regressors were included to model the 6 movement parameters and the mean for each run, with rest blocks providing an implicit baseline. For univariate analyses, additional regressors were included for standard and catch trials, as well as feedback trials for participants 6 to 24. Contrast images from the first-level model (average of all standard trials) were entered into a second-level one-sample *t* test, using participants as a random effect. For multivariate analyses, for each run, separate regressors were included for each trained item (*n* = 48), plus a regressor of no interest that included catch trials, as well as feedback trials for participants 6 to 24. T-statistic maps were generated for the contrast of each item in each run relative to the unmodeled rest period, creating 192 statistical maps. As T maps combine the effect size weighted by error variance for a modeled response, they provide high-classification accuracy in multivariate analyses, since results are not unduly influenced by large, but highly variable, response estimates (34).

Searchlight RSA was conducted on these T maps using the CoSMoMVPA toolbox (35). First, a mean T map was generated for each item, collapsed across run. Using spherical searchlights with a radius of 3 voxels (minimum 2 voxels per searchlight), data were extracted from gray-matter masked (voxels with >0.01 gray matter probability) native-space T maps. A neural DSM was then constructed for each searchlight, in which each cell represents 1 minus the Pearson product moment correlation between the voxel-wise T statistic for each pair of items. For each searchlight, the Spearman rank correlation between the neural DSM and a set of predicted DSMs was calculated. The correlation between the predicted and neural DSM for each searchlight was converted to a z value using a Fisher transform, to conform to statistical assumptions (normality) required for second-level parametric tests. This Fisher-transformed correlation coefficient was then returned to the searchlight’s central voxel. Whole-brain Fisher-transformed correlation maps were normalized to Montreal Neurological Institute (MNI) space using parameters estimated during the segmentation stage of preprocessing. Using these maps, second-level one-sample *t* tests identified voxels in which the correlation across participants between the predicted and neural DSM was greater than zero.

We also used multiple regression to assess the relative contribution of predicted DSMs to explaining variance in the neural DSM. This yields beta values at each searchlight location that express the independent variance in the neural DSM accounted for by each predicted DSM, independent of other predicted DSMs. Whole-brain beta-statistic maps were normalized to MNI space, and maps of beta values were submitted to second-level one-sample *t* tests to identify voxels in which the independent variance in the neural DSM accounted for by each predicted DSM was greater than zero.

ROI analyses were conducted in six 4-mm-radius spherical ROIs in left and right vOT based on ref. 9 (Fig. 4*B*). Mean correlation/beta values in these ROIs were extracted from whole-brain searchlight maps using MarsBaR (36). Second-level one-sample *t* tests were used to identify ROIs in which the correlation/independent beta value was greater than zero.

We used 3 predicted DSMs to test models of visual word form representation. Each included only within-orthography pairs, since no letters are shared between the 2 orthographies. The visual DSM used HMAX simple-cell representations (s1 layer; ref. 21, see also ref. 22), which were generated for the greyscale image of each item (source code at http://maxlab.neuro.georgetown.edu/hmax.html#updated). These are simulated by Gabor filters of 4 orientations (0°, 90°, −45°, 45°) and 16 sizes (7 to 37 pixels), yielding 64 simple cell maps, which were vectorized and concatenated to form a representational vector for each item. Dissimilarity was computed as 1 minus the Pearson correlation between the vectors for each item pair. The position-specific letter DSM was computed as 1 minus the proportion of letters shared in the same position for each item pair. For example, 
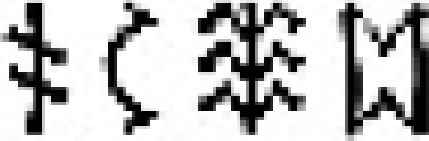
 /bεz/ and 
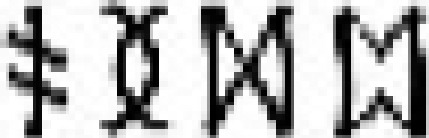
 /bəʊv/ share 2/4 letters, whereas 
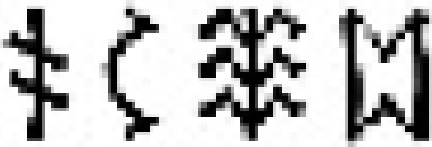
 /bεz/ and 
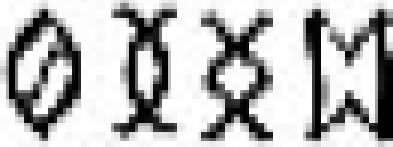
 /fəʊt/ share 1/4 letters. In the spatial coding DSM, the similarity between item pairs was graded according to the distance in position between shared letters, with additional weighting for shared beginning and/or end letters, with similarity values generated using Match Calculator (http://www.pc.rhul.ac.uk/staff/c.davis/utilities/matchcalc/index.htm). For example, 
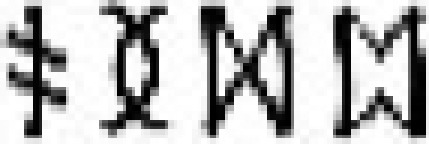
 /bəʊv/ and 
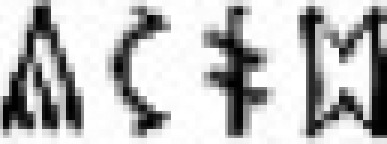
 /pεb/ have a similarity of 0.38, whereas 
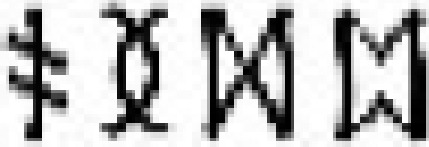
 /bəʊv/ and 
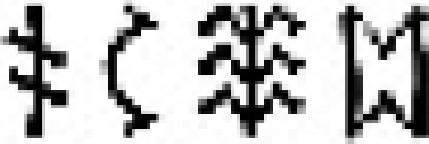
 /bεz/ have a similarity of 0.67, since /b/ is in a different position in the first pair but in the same position in the second. Dissimilarity was expressed as 1 minus these similarity values.

We also tested models of phonological and semantic similarity. Only between-orthography item pairs were included, to ensure results were independent of orthographic similarity. The phonological DSM was computed as 1 minus the proportion of same-position phonemes shared. For example, 
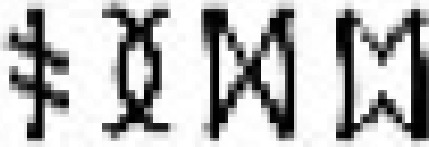
 /bəʊv/ and 
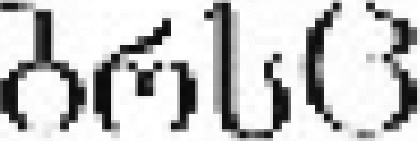
 /buv/ share 2/3 phonemes. The semantic DSM had values of zero for item pairs from the same semantic category, and 1 for pairs from different categories.

## Supplementary Material

Supplementary File
