# Supplementary material for: Mapping visual symbols onto spoken language along the ventral visual stream
Source: Proc Natl Acad Sci U S A. 2019 Aug 19;116(36):17723–8. doi: 10.1073/pnas.1818575116 (PMC6731646; doi:10.1073/pnas.1818575116)
Supplement: Supplementary File [file pnas.1818575116.sapp.pdf]

Supplementary information text

Author Note: These data were collected whilst J. S. H. Taylor was a post-doctoral fellow at Royal Holloway, University of London. Funding for this work was provided by the Economic and Social Research Council (ES/L002264/1). Matthew H. Davis was supported by the UK Medical Research Council (SUAG/008 RG91365). We are grateful to Clare Lally for research assistance.

SI Methods

Stimuli (examples in Figure 1).

Phonological forms. Two sets of 24 consonant-vowel-consonant pseudowords (henceforth, languages) were constructed from 8 consonants, /b/, /f/, /g/, /m/, /p/, /t/, /v/, /z/, and 8 vowels, 4 of which were used for each language; /ɛ/, /ʌ/, /aɪ/, /əʊ/ (language 1); /æ/, /ɒ/, /i/, /u/, (language 2). Within each language, consonants occurred three times in onset position, and three times in coda position, whereas vowels occurred six times each. Pseudowords were recorded by a female native English speaker and digitised at a sampling rate of 44.1 KHz.

Orthographic forms. Two sets of 20 alphabetic symbols were selected from two archaic orthographies (Hungarian runes, Georgian *Mkhedruli*). The 16 phonemes comprising the two languages were associated with one symbol from each orthography. The remaining 4 symbols in each orthography had no associated sound. The written form of each trained item comprised four symbols: the first three symbols associated with the phonemes in each trained word, and a final silent symbol. Written forms of the trained items from both languages were constructed using each orthography, and the assignment of language to orthography was counterbalanced across participants. Symbols varied in both height and

width and were left and bottom aligned when constructing written forms, ensuring a similar gap between symbols. Words therefore varied in width, but were centered on a white background 320 x 112 pixels.

Semantic forms. Two sets of 24 familiar objects were selected (pictures and English names). Each set comprised 6 fruits or vegetables, 6 vehicles, 6 animals, and 6 tools. For each participant, for one language (henceforth the systematic language), the semantic categories were assigned to the trained items systematically according to the final symbol (e.g. animals were assigned to items ending in one symbol, tools to another symbol, etc.). For the other language (henceforth the arbitrary language), there was an arbitrary assignment of meaning to trained item, such that there was an equal probability of each final symbol occurring with each semantic category. The assignment of orthography to the systematic or arbitrary language was counterbalanced across participants. Note that the findings reported in the current manuscript were part of a larger behavioural study concerned with the learning and generalization of spelling-to-sound and spelling-to-meaning regularities, therefore comparisons of systematic versus arbitrary orthography–semantic mappings are not reported here.[1]

Generalisation items. For each trained item, an untrained item was created that differed in either the vowel or final consonant as well as in the final silent symbol. These were used to assess extraction of symbol-sound mappings at the end of training.

---

[1] A reviewer asked whether the orthography–semantic systematicity of the final letter impacted on the results reported in the current manuscript. At the end of training, saying the meanings of the artificial written words, a task similar to that used in the scanner, was equivalent in accuracy, $t(22) = 1.10$, ns, and response times, $t(22) < 1$, ns, for the systematic (mean accuracy = 93%, RT = 2182ms) and the arbitrary (mean accuracy = 91%, RT = 2133ms) language (note that one participant misunderstood the test task and so is excluded from these analyses). Furthermore, there was no evidence from exploratory analyses of the neuroimaging data that the systematicity of the final letter enhanced the representation of letter identity or position information (correlations between neural data and position-specific and spatial coding models no greater for the systematic than the arbitrary language).

Behavioural training and testing procedure

Spoken responses for all tasks were recorded and manually coded for accuracy and RT. Keyboard and mouse accuracy and RTs were recorded by E-Prime.

Phonology-to-Semantic pre-training. Before beginning the orthography training, participants learned the spoken form to meaning associations for the 24 pseudowords from each language, using the procedure described in (1). At the end of three pre-training runs for each language they achieved good accuracy for saying the novel word to match the picture (Language 1 = 60% (SD = 22%), Language 2 = 64% (SD = 21%)), and for saying the meaning to match the heard novel word (Language 1 = 63% (SD = 22%), Language 2 = 62% (SD = 20%)).

***Orthography training*.** Participants learned about the two orthographies for ~1.5 hours per day, for nine consecutive days, with breaks for weekends. Four tasks were completed each day for each orthography and the order in which the tasks and orthographies were presented was varied across days.

i) *Reading aloud (24 trials, 4 repetitions).* The orthographic forms of each of the 24 trained items were presented in a randomised order. Participants read them aloud, i.e., said their pronunciation in the new language, and then pressed spacebar to hear the correct answer.

ii) *Saying the meaning (24 trials, 4 repetitions)*. As for reading aloud, but participants said the English meaning of each item aloud.

iii) *Orthographic search (48 trials).* Participants saw a picture of one of the trained meanings and used the mouse to select the orthographic form that matched it from all 24 trained items. They then did this task in reverse, i.e. a single orthographic form was

displayed and they picked the correct picture out of 24. On each trial they received feedback indicating the correct item.

iv) Meaning judgement (72 trials). Participants saw the orthographic forms of four trained items, two from each of two semantic categories, and a sentence describing one of the items. The sentence described what the item looked like (24 trials), its function (24 trials), or its location (animals, tools, vehicles) or taste (vegetables/fruit). Each trained item appeared six times either as a target or same-category distractor. Other-category distractors were randomly selected on each trial. Participants used the numbers 1 to 4 on the keyboard to select which item was described by the sentence, and received feedback as to the correct item.

Post-test procedure. The day after the final day of training, participants completed several tasks, three of which are relevant to the current manuscript since they tested their learning of the trained items and generalization to untrained items. Each task was completed for both orthographies.

i) Reading aloud (24 trials). The orthographic forms of each of the 24 trained items were presented in a randomised order. Participants read them aloud, i.e., said their pronunciation in the new language, and then pressed spacebar to move onto the next item.

ii) Saying the meaning (24 trials). As for reading aloud, but participants said the English meaning of each item aloud.

iii) Generalization (24 trials). As for reading aloud, but participants were presented with untrained items and said their pronunciation.

Functional imaging acquisition. Functional MRI data were acquired in two scan sessions on separate days on a 3T Siemens Trio scanner (Siemens Medical Systems, Erlangen, Germany) with a 32-channel head coil. Blood oxygenation level-dependent fMRI images were acquired with fat saturation, 3mm isotropic voxels consisting of 32 x 3mm slices with an interslice gap of .75mm, flip angle of 78 degrees, echo time [TE] = 30 ms, and a 64 x 64 data matrix. We used a continuous acquisition sequence with a 2000ms repetition (TR) and acquisition (TA) time. Acquisition was transverse oblique, angled to avoid the eyes and to achieve whole-brain coverage including the cerebellum. In a few cases the top of the parietal lobe was not covered. In each scan session a T_1-weighted structural volume was also acquired using a magnetization prepared rapid acquisition gradient echo protocol (TR = 2250 ms, TE = 2.99 ms, flip angle = 9 degrees, 1mm slice thickness, 256x 240 x 192 matrix, resolution = 1 mm isotropic).

Two runs were collected on each day and in each run, 438 images were acquired. Image processing and statistical analyses were performed using SPM8 (Wellcome Trust Centre for Functional Neuroimaging, London, UK). The first 6 volumes of each run were discarded to allow for equilibration effects. Slice timing correction was applied, referenced to the middle slice. Images for each participant were realigned to the first image in the run (2). For univariate analyses, images were coregistered to the structural image collected on the same day as scanning prior to normalization. For multivariate analyses, all functional images were coregistered to the structural image collected on the first scan day, since subsequent analyses of these data were conducted in native space (3). For both uni and multivariate analyses, the origin of all functional and structural images were then manually registered to the anterior commissure. The transformation required to bring a participant's structural T1 images into standard Montreal Neurological Institute (MNI) space was

calculated using tissue probability maps (4). For univariate analyses, these warping parameters were applied to all functional images for that participant. Normalised functional images were then re-sampled to 2mm isotropic voxels and the data were spatially smoothed with 8mm full-width half maximum isotropic Gaussian kernel prior to model estimation. For multivariate analyses we used unsmoothed native space images.

Data from each participant were entered into general linear models for event-related analysis (5). In all models, events were convolved with the SPM8 canonical hemodynamic response function. Movement parameters estimated at the realignment stage of pre-processing were added as regressors of no interest in addition to the session mean. Low frequency drifts were removed with a high-pass filter (128s) and AR1 correction for serial autocorrelation was made.

SI Results

Justification of multiple regression method

We used multiple regression rather than partial correlation because we sought to determine the independent variance in the neural response patterns explained by either/both DSMs across each region. Multicollinearity diagnostics indicated that the correlation between the similarity values for the two coding schemes was not too high to justify using multiple regression methods, Spearman $r(552) = .86$, $VIF = 4.07$, i.e. $VIF < 10$.

Open-bigram coding analysis

We conducted a similar analysis using a predicted DSM derived from open-bigram coding (6), in which similarity between items depends on shared contiguous or non-contiguous same order letter pairs (values generated using Match Calculator). Multicollinearity

diagnostics for the position-specific and open-bigram coding DSMs were, $r(552) = .61$, VIF = 1.58. Results (SI Appendix, Table S6, Figure S1, S2) were broadly similar to the analysis including position-specific and spatial coding predicted DSMs (although open-bigram coding accounted for independent variance in fewer of the right hemisphere vOT ROIs).

SI Discussion

The present experiment did not attempt to dissociate sensitivity to within-word position from sensitivity to retinal location since written words were always presented at fixation. However, these factors were not entirely confounded, because letters varied in width and words were constructed to ensure a similar gap between letters. Therefore, words also varied in width. Consequently, retinal location was not exactly the same even for the same letter in the same within-word position. Thus, even correlations between the position-specific letter DSM and the neural response patterns may indicate a degree of tolerance to retinal location shifts.

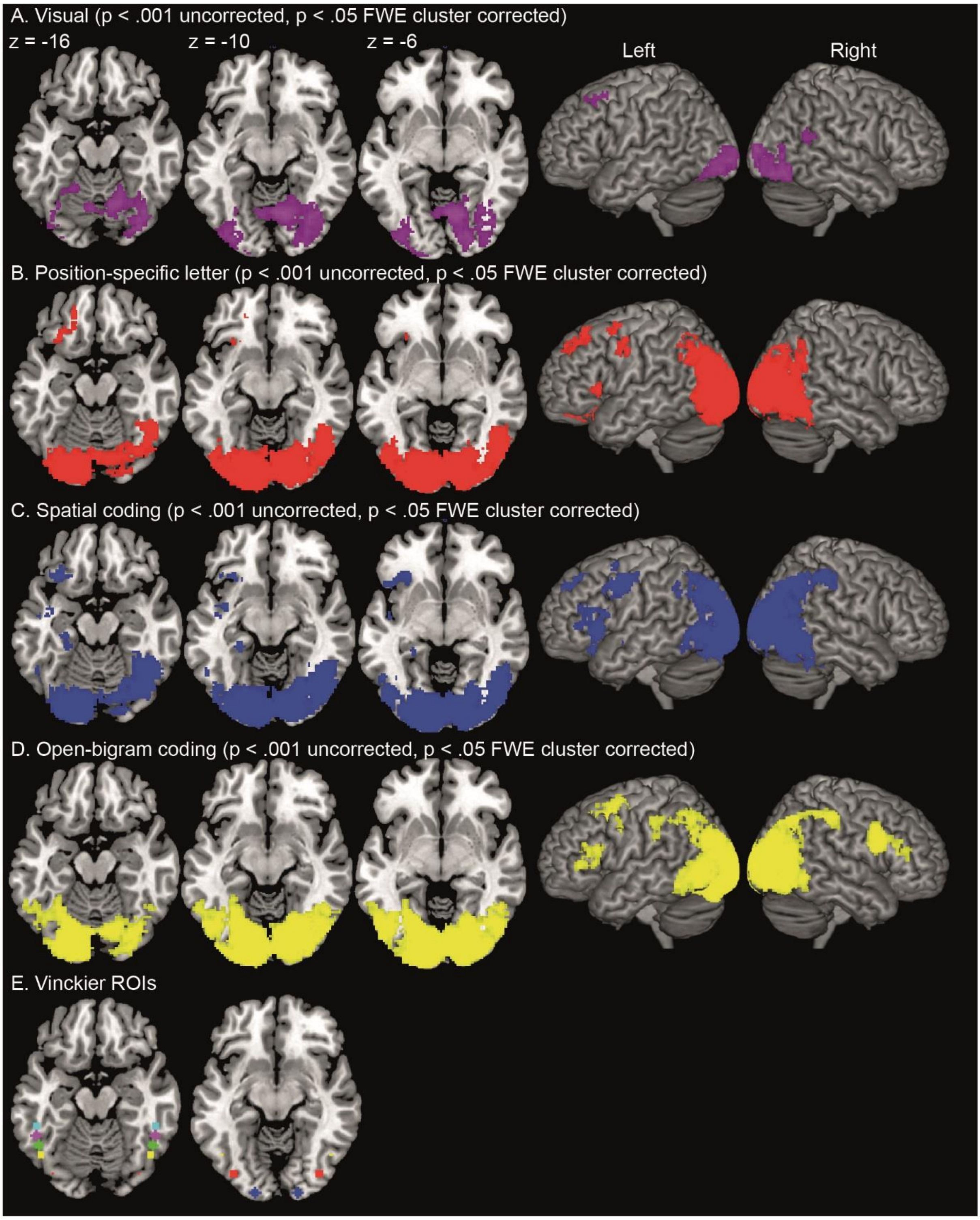

Figure S1.

Brain regions in which second-level one-sample t-tests demonstrated that the neural DSM was positively correlated with several different within-orthography predicted DSMs, across 24 participants, at a threshold of $p < .001$ uncorrected, $p < .05$ FWE cluster corrected. Axial slices are shown as well as left and right hemisphere renderings and panel E shows the

position of the Vinckier ROIs in the same axial slices. A) visual DSM (s1 layer representations from HMAX, B) position-specific letter DSM, C) spatial-coding DSM, D) open-bigram coding DSM.

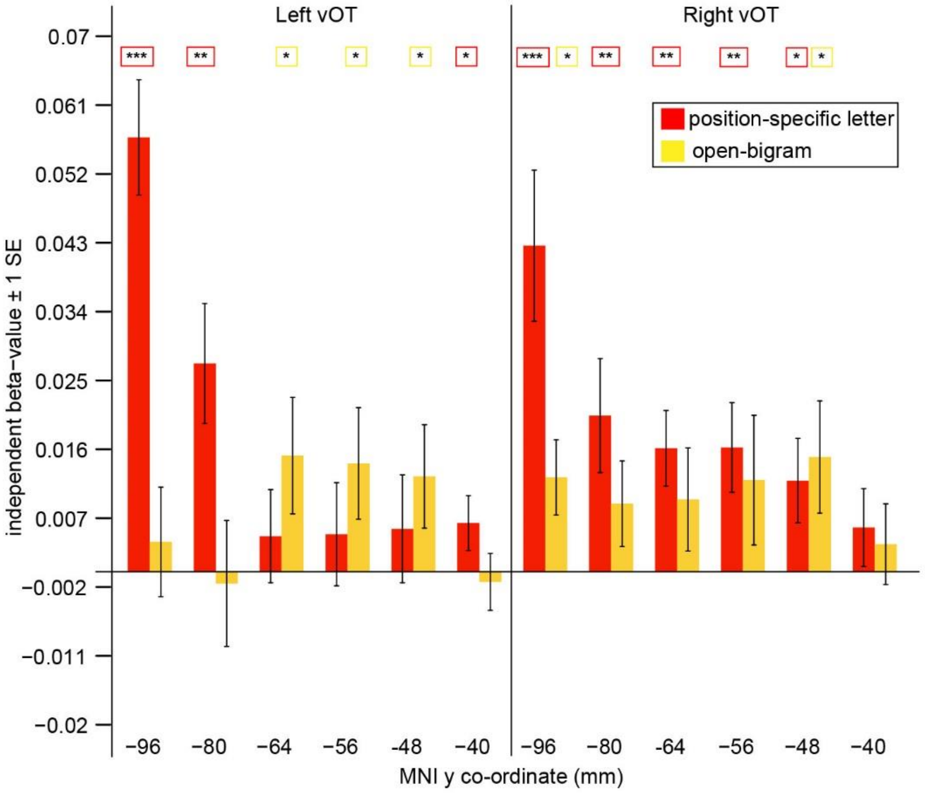

Figure S2.

Results of a simultaneous multiple regression analysis examining the independent variance accounted for by position-specific letter coding versus open-bigram coding (6). In the open-bigram coding DSM, each cell represented one minus the similarity of each item pair (within the same orthography) according to open-bigram coding, generated using Match Calculator. Along the x-axis, ROIs in the left and right hemisphere go from posterior to anterior vOT. Red and yellow bars show the mean independent beta-value for the position-specific letter and open-bigram coding DSMs, collapsed across subjects. Statistics above the bars denote whether second level one-sample t-tests on the resulting beta-values for each DSM in each of the ROIs were significantly greater than zero (one-tailed t-test, *** = $p < .001$, ** = $p < .01$, * = $p < .05$). Standard error bars are appropriate for these one-sample t-tests.

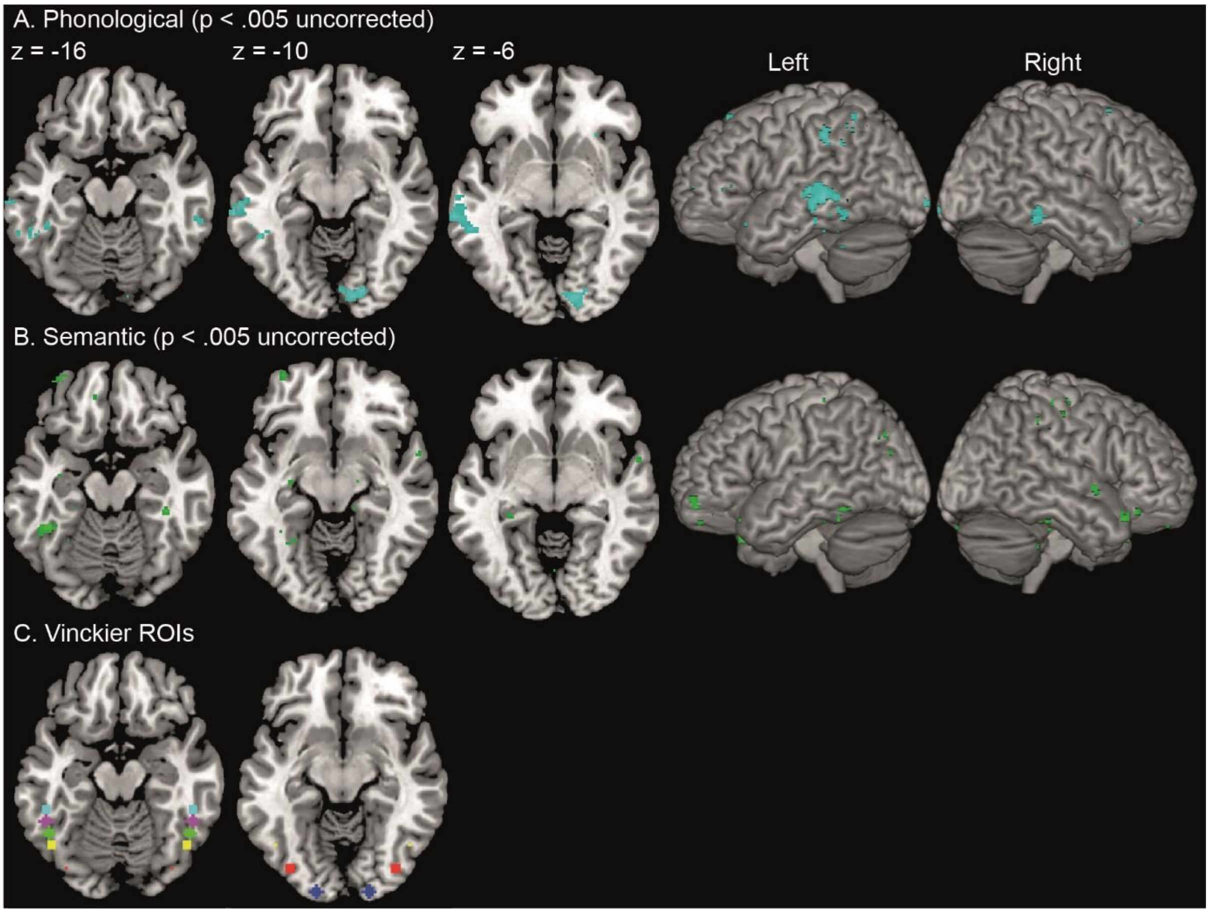

Figure S3.

Brain regions in which second-level one-sample t-tests demonstrated that the neural DSM was positively correlated with two between-orthography predicted DSMs, across 24 participants, at a threshold of p < .005 uncorrected. Axial slices are shown as well as left and right hemisphere renderings and panel C shows the position of the Vinckier ROIs in the same axial slices. A) phonological DSM, B) semantic category DSM.

Table S1

Brain regions active when viewing learned words, relative to un-modelled resting baseline for 24 participants. Top 20 peaks > 8mm apart are reported at a threshold of p < .001 uncorrected, and p < .05 FWE cluster corrected. Bold text denotes the first peak within a cluster. Anatomical labels in this and all subsequent tables were generated using the automated anatomical labelling template (7) toolbox implemented in SPM8.

Location	Hemisphere	X	Y	Z	No. voxels	Z-value	Cluster p-value
**Middle Occipital Gyrus**	**Left**	**-30**	**-94**	**-2**	**2884**	**Inf**	**< .001**
Lingual Gyrus	Left	-16	-92	-12			
Inferior Occipital Gyrus	Left	-40	-74	-6			
**Middle Occipital Gyrus**	**Right**	**30**	**-90**	**4**	**2620**	**7.74**	**< .001**
Calcarine Gyrus	Right	24	-94	0			
Inferior Occipital Gyrus	Right	40	-76	-12			
Inferior Occipital Gyrus	Right	42	-68	-10			
Cerebelum VI	Right	30	-62	-26			
**Superior Parietal Lobule**	**Left**	**-24**	**-66**	**40**	**1083**	**5.94**	**< .001**
Inferior Parietal Lobule	Left	-28	-54	40			
**Precentral Gyrus**	**Left**	**-42**	**4**	**32**	**1417**	**5.27**	**< .001**
IFG p. Triangularis	Left	-48	30	20			
Precentral Gyrus	Left	-54	-2	44			
Precentral Gyrus	Left	-46	-2	52			
**Posterior-Medial Frontal**	**Left**	**-4**	**4**	**64**	**337**	**5.05**	**0.035**

Table S2

Brain regions in which a second-level one-sample t-test demonstrated that the neural DSM was positively correlated with a within-orthography basic visual (s1 layer representations from the HMAX model) predicted DSM, across 24 participants. Top 20 peaks > 8mm apart are reported at a threshold of $p < .001$ uncorrected, and $p < .05$ FWE cluster corrected. Bold text denotes the first peak within a cluster.

Location	Hemisphere	X	Y	Z	No. voxels	z-value	Cluster p-value
**Cerebellum**	**Right**	**4**	**-66**	**-8**	**3678**	**4.95**	**< .001**
Calcarine Gyrus	Right	12	-78	8			
Calcarine Gyrus	Right	20	-90	-2			
Lingual Gyrus	Right	12	-68	-2			
Calcarine Gyrus	Right	18	-74	4			
Inferior Occipital Gyrus	Right	36	-82	-14			
Superior Occipital Gyrus	Right	22	-96	6			
Lingual Gyrus	Right	10	-76	-2			
Fusiform Gyrus	Right	36	-72	-14			
Inferior Occipital Gyrus	Right	40	-66	-10			
Middle Occipital Gyrus	Right	38	-74	2			
Cerebelum VI	Left	-8	-68	-14			
Fusiform Gyrus	Right	30	-76	-6			
Middle Occipital Gyrus	Right	28	-88	-2			
Cerebelum IV-V	Right	18	-56	-16			
Inferior Occipital Gyrus	Right	28	-90	-12			
Inferior Temporal Gyrus	Right	38	-56	-8			
White Matter	Right	32	-64	-6			
Cuneus	Right	12	-82	24			
Cerebellum	Left	-4	-68	-4			
**Middle Occipital Gyrus**	**Left**	**-28**	**-96**	**-2**	**848**	**4.23**	**< .001**
Inferior Occipital Gyrus	Left	-32	-88	-10			
Fusiform Gyrus	Left	-34	-66	-18			
Middle Occipital Gyrus	Left	-24	-100	6			
Inferior Occipital Gyrus	Left	-34	-80	-12			
Inferior Occipital Gyrus	Left	-34	-74	-6			
Middle Occipital Gyrus	Left	-26	-92	8			
Inferior Occipital Gyrus	Left	-20	-100	-8			
Middle Occipital Gyrus	Left	-18	-92	0			
Fusiform Gyrus	Left	-30	-64	-10			
Inferior Occipital Gyrus	**Left**	-48	-80	-16			
Middle Occipital Gyrus	Left	-38	-90	6			
Cerebellum Crus 1	Left	-42	-72	-20			
**Superior Temporal Gyrus**	**Right**	**64**	**-54**	**24**	**153**	**4**	**.002**
Middle Temporal Gyrus	Right	54	-52	18			

Location	Hemisphere	X	Y	Z	No. voxels	z-value	Cluster p-value
Superior Temporal Gyrus	Right	44	-44	18			
**Middle Frontal Gyrus**	**Left**	**-30**	**22**	**46**	**161**	**3.87**	**.001**
Middle Frontal Gyrus	Left	-34	18	54			
Superior Frontal Gyrus	Left	-16	28	48			

Table S3

Brain regions in which a second-level one-sample t-test demonstrated that the neural DSM was positively correlated with a within-orthography position-specific letter predicted DSM, across 24 participants. Top 20 peaks > 8mm apart are reported at a threshold of p < .001 uncorrected, and p < .05 FWE cluster corrected. Bold text denotes the first peak within a cluster.

Location	Hemisphere	X	Y	Z	No. voxels	Z-value	Cluster p-value
**Middle Occipital Gyrus**	**Left**	**-30**	**-74**	**28**	**18440**	**6.59**	**< .001**
Calcarine Gyrus	Left	-10	-94	-4			
Calcarine Gyrus	Left	-2	-92	8			
Lingual Gyrus	Left	-18	-86	-12			
Superior Parietal Lobule	Left	-22	-62	44			
Inferior Occipital Gyrus	Left	-18	-96	-10			
Calcarine Gyrus	Left	-6	-86	-10			
Calcarine Gyrus	Right	10	-86	2			
Calcarine Gyrus	Right	14	-92	-4			
Calcarine Gyrus	Right	8	-90	10			
Superior Occipital Gyrus	Left	-12	-94	8			
Lingual Gyrus	Right	10	-84	-6			
Middle Occipital Gyrus	Right	46	-74	0			
Inferior Occipital Gyrus	Left	-24	-92	-6			
Middle Temporal Gyrus	Right	46	-64	2			
Superior Occipital Gyrus	Right	24	-66	42			
Middle Occipital Gyrus	Left	-20	-96	2			
Superior Occipital Gyrus	Right	22	-98	10			
Lingual Gyrus	Right	16	-78	-4			
Superior Occipital Gyrus	Left	-18	-86	20			
**Superior Frontal Gyrus**	**Left**	**-14**	**32**	**54**	**524**	**4.51**	**< .001**
Superior Frontal Gyrus	Left	-22	42	38			
Superior Frontal Gyrus	Left	-14	42	42			
Middle Frontal Gyrus	Left	-20	26	52			
Middle Frontal Gyrus	Left	-30	30	48			
Middle Frontal Gyrus	Left	-22	30	42			
Middle Frontal Gyrus	Left	-20	40	28			
Superior Frontal Gyrus	Left	-16	50	40			
**Superior Orbital Gyrus**	**Left**	**-18**	**42**	**-14**	**252**	**4.05**	**< .001**
IFG p. Orbitalis	Left	-30	24	-16			
Superior Orbital Gyrus	Left	-16	32	-18			
IFG p. Triangularis	Left	-34	32	4			
Rectal Gyrus	Left	-8	36	-22			
Insula Lobe	Left	-30	26	-6			
White Matter	Left	-24	22	-10			
**Precentral Gyrus**	**Left**	**-46**	**-4**	**48**	**107**	**3.93**	**0.007**
Postcentral Gyrus	Left	-50	-6	38			
Precentral Gyrus	Left	-54	0	44			

Location	Hemisphere	X	Y	Z	No. voxels	Z-value	Cluster p-value
**Middle Frontal Gyrus**	**Left**	**-26**	**8**	**52**	**204**	**3.85**	**< .001**
Precentral Gyrus	Left	-32	2	60			
Middle Frontal Gyrus	Left	-24	6	62			
Superior Frontal Gyrus	Left	-22	-2	54			
**IFG p. Triangularis**	**Left**	**-50**	**20**	**6**	**95**	**3.63**	**0.014**
IFG p. Triangularis	Left	-56	26	4			

Table S4

Brain regions in which a second-level one-sample t-test demonstrated that the neural DSM was positively correlated with a within-orthography spatial-code DSM, across 24 participants. Top 20 peaks > 8mm apart are reported at a threshold of p < .001 uncorrected, and p < .05 FWE cluster corrected. Bold text denotes the first peak within a cluster.

Location	Hemisphere	X	Y	Z	No. voxels	Z-value	Cluster p-value
**Calcarine Gyrus**	**Left**	**-8**	**-92**	**-2**	**20298**	**6.96**	**< .001**
Calcarine Gyrus	Left	-2	-92	6			
Calcarine Gyrus	Left	-6	-86	-10			
Calcarine Gyrus	Right	8	-86	0			
Lingual Gyrus	Left	-18	-86	-12			
Lingual Gyrus	Right	14	-90	-4			
Inferior Temporal Gyrus	Right	48	-72	-10			
Lingual Gyrus	Left	-6	-82	-2			
Middle Occipital Gyrus	Left	-28	-72	26			
Superior Occipital Gyrus	Left	-12	-96	8			
Area hOc3v [V3v]	Left	-20	-84	-4			
Lingual Gyrus	Right	20	-80	-8			
Inferior Occipital Gyrus	Left	-24	-92	-6			
Inferior Temporal Gyrus	Right	42	-66	-6			
Superior Occipital Gyrus	Right	28	-68	40			
Superior Occipital Gyrus	Right	26	-94	12			
Inferior Occipital Gyrus	Right	42	-64	-14			
Middle Occipital Gyrus	Right	30	-86	4			
Superior Parietal Lobule	Left	-24	-56	48			
Calcarine Gyrus	Right	24	-90	0			
**IFG p. Triangularis**	**Left**	**-36**	**30**	**2**	**1182**	**4.79**	**< .001**
IFG p. Triangularis	Left	-46	18	-2			
IFG p. Orbitalis	Left	-32	24	-16			
IFG p. Triangularis	Left	-48	26	16			
IFG p. Triangularis	Left	-36	28	14			
IFG p. Triangularis	Left	-52	24	4			
IFG p. Orbitalis	Left	-30	28	-4			
IFG p. Opercularis	Left	-50	16	16			
Insula Lobe	Left	-30	24	10			
IFG p. Triangularis	Left	-60	18	8			
IFG p. Orbitalis	Left	-42	22	-8			
Insula Lobe	Left	-24	22	-12			
IFG p. Triangularis	Left	-46	34	18			
IFG p. Triangularis	Left	-54	34	10			
IFG p. Triangularis	Left	-32	38	10			
Insula Lobe	Left	-32	20	-2			
Middle Frontal Gyrus	Left	-30	40	18			
IFG p. Triangularis	Left	-46	36	4			

Location	Hemisphere	X	Y	Z	No. voxels	Z-value	Cluster p-value
**Precentral Gyrus**	**Left**	**-48**	**0**	**38**	**661**	**4.43**	**< .001**
IFG p. Opercularis	Left	-40	16	34			
Precentral Gyrus	Left	-38	0	26			
Middle Frontal Gyrus	Left	-50	12	40			
Precentral Gyrus	Left	-56	8	30			
Postcentral Gyrus	Left	-60	-16	44			
**ParaHippocampal Gyrus**	**Left**	**-28**	**-24**	**-24**	**169**	**3.98**	**< .001**
ParaHippocampal Gyrus	Left	-24	-36	-12			
ParaHippocampal Gyrus	Left	-28	-28	-16			
**Middle Frontal Gyrus**	**Left**	**-24**	**8**	**46**	**154**	**3.83**	**0.001**
Middle Frontal Gyrus	Left	-36	4	58			
Middle Frontal Gyrus	Left	-26	8	54			
**Insula Lobe**	**Left**	**-38**	**-2**	**-10**	**105**	**3.76**	**0.01**
Area Id1	Left	-42	-10	-14			
Superior Temporal Gyrus	Left	-46	-4	-6			
**Superior Frontal Gyrus**	**Left**	**-22**	**42**	**38**	**293**	**3.71**	**< .001**
Superior Frontal Gyrus	Left	-18	32	48			
Superior Frontal Gyrus	Left	-12	22	54			
Superior Frontal Gyrus	Left	-14	50	38			
Superior Frontal Gyrus	Left	-14	42	42			

Table S5

Results of second-level one-sample t-tests on Fisher transformed Spearman rank correlations between predicted and neural DSMs. For each participant, correlations were extracted from left and right hemisphere vOT ROIs following whole-brain searchlight analyses. The predicted DSMs are a visual model computed using the simple cell representations from the HMAX model (1 minus correlation between s1 layer representations of item pairs), a position-specific letter model (1 – proportion of same-position letters shared between items), and a more position-invariant letter model (1 – spatial code similarity). Correlations that are significantly greater than zero (one-tailed t-test) are shown in bold.

		ROI Location					
		±18 -96 -10	±36 -80 -12	±46 -64 -14	±48 -56 -16	±50 -48 -16	±50 -40 -18
Left vOT		**Basic Visual**					
	r(23)	**0.035**	**0.065**	0.015	0.017	0.019	0.004
	t-stat	**2.71**	**4.359**	1.129	1.177	1.481	0.499
	SE	**0.013**	**0.015**	0.013	0.014	0.013	0.008
	pval	**0.006**	**< .001**	0.135	0.126	0.076	0.311
		**Letters**					
	r(23)	**0.054**	**0.029**	**0.012**	**0.015**	**0.014**	**0.006**
	t-stat	**8.585**	**4.898**	**3.075**	**2.516**	**2.162**	**2.045**
	SE	**0.006**	**0.006**	**0.004**	**0.006**	**0.006**	**0.003**
	pval	**< .001**	**< .001**	**0.003**	**0.01**	**0.021**	**0.026**
		**Spatial**					
	r(23)	**0.049**	**0.027**	**0.019**	**0.021**	**0.019**	**0.006**
	t-stat	**7.535**	**4.53**	**3.528**	**3.369**	**2.887**	**2.483**
	SE	**0.007**	**0.006**	**0.005**	**0.006**	**0.006**	**0.003**
	pval	**< .001**	**< .001**	**0.001**	**0.001**	**0.004**	**0.01**
Right vOT		**Basic Visual**					
	r(23)	**0.071**	**0.085**	**0.053**	**0.042**	**0.038**	0.002
	t-stat	**3.411**	**4.858**	**2.725**	**2.297**	**2.826**	0.165
	SE	**0.021**	**0.017**	**0.019**	**0.018**	**0.013**	0.011
	pval	**0.001**	**< .001**	**0.006**	**0.016**	**0.005**	0.435
		**Letters**					
	r(23)	**0.052**	**0.027**	**0.019**	**0.021**	**0.02**	**0.008**
	t-stat	**4.779**	**4.026**	**5.588**	**5.015**	**3.987**	**1.874**
	SE	**0.011**	**0.007**	**0.003**	**0.004**	**0.005**	**0.005**
	pval	**< .001**	**< .001**	**< .001**	**< .001**	**< .001**	**0.037**
		**Spatial**					
	r(23)	**0.037**	**0.025**	**0.026**	**0.026**	**0.024**	**0.011**
	t-stat	**4.733**	**4.476**	**5.582**	**5.341**	**4.13**	**2.651**
	SE	**0.008**	**0.006**	**0.005**	**0.005**	**0.006**	**0.004**
	pval	**< .001**	**< .001**	**< .001**	**< .001**	**< .001**	**0.007**

Table S6

Brain regions in which a second-level one-sample t-test demonstrated that the neural DSM was positively correlated with a within-orthography open-bigram coding DSM, across 24 participants. Top 20 peaks > 8mm apart are reported at a threshold of p < .001 uncorrected, and p < .05 FWE cluster corrected. Bold text denotes the first peak within a cluster.

Location	Hemisphere	X	Y	Z	No. voxels	Z-value	Cluster p-value
**Calcarine Gyrus**	**Left**	**-10**	**-88**	**0**	**21275**	**6.87**	**< .001**
Calcarine Gyrus	Left	-2	-90	-2			
Calcarine Gyrus	Left	-6	-86	-10			
Middle Occipital Gyrus	Left	-18	-90	-8			
Lingual Gyrus	Right	12	-82	0			
Calcarine Gyrus	Right	12	-90	4			
Cuneus	Right	12	-98	6			
Fusiform Gyrus	Right	26	-74	-6			
Middle Occipital Gyrus	Right	30	-86	4			
Middle Occipital Gyrus	Right	32	-82	24			
Superior Occipital Gyrus	Left	-12	-94	8			
Superior Occipital Gyrus	Right	26	-94	12			
Fusiform Gyrus	Left	-22	-82	-12			
Middle Occipital Gyrus	Left	-20	-96	-2			
Angular Gyrus	Right	26	-60	42			
Lingual Gyrus	Left	-20	-74	-12			
Middle Occipital Gyrus	Right	28	-86	18			
Middle Occipital Gyrus	Right	30	-74	20			
Inferior Occipital Gyrus	Right	26	-90	-2			
Inferior Occipital Gyrus	Left	-50	-70	-4			
**IFG p. Opercularis**	**Right**	**48**	**10**	**22**	**989**	**4.82**	**< .001**
IFG p. Opercularis	Right	42	8	32			
IFG p. Opercularis	Right	48	14	30			
RPrecentral Gyrus	Right	50	8	36			
Middle Frontal Gyrus	Right	38	30	22			
IFG p. Triangularis	Right	46	30	24			
IFG p. Triangularis	Right	54	28	14			
IFG p. Triangularis	Right	50	36	16			
RPrecentral Gyrus	Right	58	6	36			
IFG p. Triangularis	Right	48	22	26			
**Middle Frontal Gyrus**	**Left**	**-28**	**10**	**48**	**1110**	**4.67**	**< .001**
Middle Frontal Gyrus	Left	-28	18	42			
Superior Frontal Gyrus	Left	-20	28	46			
Superior Frontal Gyrus	Left	-20	0	60			
Precentral Gyrus	Left	-30	-2	58			
Middle Frontal Gyrus	Left	-22	2	52			
Middle Frontal Gyrus	Left	-36	6	52			
IFG p. Opercularis	Left	-36	8	26			

Location	Hemisphere	X	Y	Z	No. voxels	Z-value	Cluster p-value
Superior Frontal Gyrus	Left	-16	24	52			
Precentral Gyrus	Left	-46	0	34			
Superior Frontal Gyrus	Left	-18	10	56			
Precentral Gyrus	Left	-40	4	42			
White Matter	Left	-24	-6	44			
Middle Frontal Gyrus	Left	-38	10	34			
Middle Frontal Gyrus	Left	-24	22	56			
Precentral Gyrus	Left	-48	2	46			
Precentral Gyrus	Left	-40	0	28			
**IFG p. Triangularis**	**Left**	**-52**	**38**	**4**	**438**	**3.79**	**< .001**
IFG p. Triangularis	Left	-54	22	18			
IFG p. Triangularis	Left	-46	22	6			
IFG p. Triangularis	Left	-44	28	14			
IFG p. Triangularis	Left	-34	28	10			
IFG p. Triangularis	Left	-42	32	22			
IFG p. Triangularis	Left	-56	30	12			
IFG p. Triangularis	Left	-46	20	16			
IFG p. Triangularis	Left	-58	22	10			
**SupraMarginal Gyrus**	**Left**	**-58**	**-30**	**40**	**158**	**3.58**	**0.001**
Inferior Parietal Lobule	Left	-54	-36	44			
SupraMarginal Gyrus	Left	-66	-30	28			
SupraMarginal Gyrus	Left	-66	-34	38			

Table S7

Brain regions in which a second-level one-sample t-test demonstrated that the neural DSM was positively correlated with a between-orthography phonological DSM, across 24 participants. Top 20 peaks > 8mm apart are reported at a threshold of p < .005 uncorrected. Bold text denotes the first peak within a cluster.

Location	Hemisphere	X	Y	Z	No. voxels	z-value	Voxel p-value
**Mid-Cingulate Cortex**	**Right**	**8**	**-36**	**26**	**90**	**3.65**	**<.001**
Mid-Cingulate Cortex	Right	4	-36	36			<.001
Mid-Cingulate Cortex	Left	-2	-34	26			0.005
**Lingual Gyrus**	**Right**	**12**	**-88**	**-8**	**194**	**3.48**	**<.001**
Calcarine Gyrus	Right	12	-86	2			0.003
**Middle Temporal Gyrus**	**Left**	**-68**	**-32**	**-8**	**245**	**3.48**	**<.001**
Middle Temporal Gyrus	Left	-56	-40	-8			0.001
Middle Temporal Gyrus	Left	-64	-22	-2			0.001
Middle Temporal Gyrus	Left	-68	-24	-14			0.001
**White Matter**	**Left**	**-16**	**-26**	**34**	**76**	**3.35**	**<.001**
White Matter	Left	-10	-20	32			0.002
**Inferior Temporal Gyrus**	**Left**	**-50**	**-48**	**-10**	**45**	**3.27**	**0.001**
Inferior Temporal Gyrus	Left	-42	-38	-14			0.001
Inferior Temporal Gyrus	Left	-50	-46	-18			0.002
**Superior Frontal Gyrus**	**Left**	**-18**	**12**	**46**	**20**	**3.18**	**0.001**
**Inferior Temporal Gyrus**	**Right**	**56**	**-38**	**-20**	**42**	**3.14**	**0.001**
Inferior Temporal Gyrus	Right	62	-32	-20			0.002
**Inferior Parietal Lobule**	**Left**	**-52**	**-32**	**38**	**53**	**3.13**	**0.001**
**Calcarine Gyrus**	**Left**	**-14**	**-102**	**-2**	**11**	**2.99**	**0.001**
**White Matter**	**Right**	**6**	**-30**	**22**	**8**	**2.95**	**0.002**
**Inferior Temporal Gyrus**	**Left**	**-52**	**-42**	**-16**	**11**	**2.94**	**0.002**
**Superior Parietal Lobule**	**Left**	**-54**	**-62**	**46**	**3**	**2.93**	**0.002**
**Thalamus**	**Right**	**8**	**-28**	**2**	**26**	**2.93**	**0.002**
Thalamus	Right	18	-26	8			0.002
**Cerebelum VIII**	**Right**	**10**	**-70**	**-38**	**17**	**2.93**	**0.002**
**Postcentral Gyrus**	**Left**	**-42**	**-12**	**36**	**9**	**2.91**	**0.002**
**Fusiform Gyrus**	**Right**	**26**	**-78**	**-2**	**2**	**2.9**	**0.002**
**White Matter**	**Left**	**-16**	**-6**	**58**	**2**	**2.88**	**0.002**
**Fusiform Gyrus**	**Left**	**-38**	**-44**	**-24**	**5**	**2.88**	**0.002**
**Cerebelum VI**	**Left**	**-26**	**-50**	**-30**	**23**	**2.84**	**0.002**
**White Matter**	**Right**	**10**	**-46**	**-28**	**15**	**2.83**	**0.002**
**Superior Parietal Lobule**	**Left**	**-24**	**-46**	**48**	**2**	**2.83**	**0.002**
**Superior Frontal Gyrus**	**Left**	**-16**	**34**	**56**	**3**	**2.83**	**0.002**
**Inferior Parietal Lobule**	**Left**	**-34**	**-54**	**54**	**5**	**2.83**	**0.002**
**Inferior Parietal Lobule**	**Left**	**-42**	**-52**	**52**	**7**	**2.82**	**0.002**
**SupraMarginal Gyrus**	**Left**	**-50**	**-46**	**32**	**3**	**2.79**	**0.003**
**Inferior Temporal Gyrus**	**Left**	**-62**	**-44**	**-18**	**3**	**2.78**	**0.003**
**Middle Frontal Gyrus**	**Right**	**48**	**12**	**54**	**1**	**2.76**	**0.003**

Location	Hemisphere	X	Y	Z	No. voxels	z-value	Voxel p-value
White Matter	Left	-12	-28	28	1	2.75	0.003
White Matter	Left	-20	-42	18	12	2.75	0.003
White Matter	Left	-6	-30	22	1	2.73	0.003
Inferior Temporal Gyrus	Left	-54	-26	-22	3	2.73	0.003
IFG p. Orbitalis	Right	34	34	-20	1	2.73	0.003
White Matter	Right	28	24	-6	1	2.71	0.003
IFG p. Triangularis	Right	34	20	28	2	2.71	0.003
Middle Frontal Gyrus	Left	-22	4	48	5	2.7	0.003
White Matter	Right	6	-30	12	3	2.69	0.004
Posterior Cingulate Cortex	Right	6	-44	28	3	2.69	0.004
White Matter	Right	12	-34	18	1	2.69	0.004
IFG p. Triangularis	Left	-52	38	0	1	2.68	0.004
Inferior Parietal Lobule	Left	-52	-52	42	1	2.67	0.004
Inferior Parietal Lobule	Left	-46	-48	46	1	2.67	0.004
Superior Frontal Gyrus	Right	16	20	52	3	2.67	0.004
Angular Gyrus	Left	-32	-60	36	3	2.67	0.004
White Matter	Left	-6	-10	-8	2	2.66	0.004
Middle Frontal Gyrus	Left	-40	58	4	1	2.66	0.004
White Matter	Right	16	30	0	1	2.66	0.004
Mid-Cingulate Cortex	Right	0	-24	38	1	2.64	0.004
Temporal Pole	Left	-40	22	-22	1	2.63	0.004
Lingual Gyrus	Right	14	-78	-4	1	2.63	0.004
Superior Temporal Gyrus	Right	70	-8	8	1	2.63	0.004
White Matter	Right	14	-36	20	1	2.62	0.004
Cerebelum Crus 1	Left	-52	-44	-40	2	2.62	0.004
Middle Frontal Gyrus	Right	38	14	58	2	2.62	0.004
White Matter	Left	-38	-20	26	1	2.62	0.004
Middle Occipital Gyrus	Left	-32	-64	32	1	2.61	0.004
Middle Temporal Gyrus	Left	-52	-36	-16	2	2.61	0.005
Middle Temporal Gyrus	Right	50	-36	-14	1	2.61	0.005
Fusiform Gyrus	Left	-36	-32	-26	1	2.6	0.005
Medial Temporal Pole	Right	44	22	-36	1	2.6	0.005
IFG p. Triangularis	Left	-44	32	4	1	2.59	0.005
IFG p. Orbitalis	Right	34	34	-8	1	2.58	0.005

Table S8
Brain regions in which a second-level one-sample t-test demonstrated that the neural DSM was positively correlated with a between-orthography semantic category DSM, across 24 participants. Top 20 peaks > 8mm apart are reported at a threshold of p < .005 uncorrected. Bold text denotes the first peak within a cluster.

Location	Hemisphere	X	Y	Z	No. voxels	z-value	Voxel p-value
**Fusiform Gyrus**	**Right**	**38**	**-34**	**-18**	**37**	**3.85**	**<.001**
**Inferior Temporal Gyrus**	**Left**	**-40**	**-44**	**-18**	**100**	**3.47**	**<.001**
Fusiform Gyrus	Left	-40	-42	-26			0.002
**Cerebelum VI**	**Right**	**20**	**-60**	**-26**	**39**	**3.31**	**<.001**
Cerebelum VI	Right	14	-66	-26			0.001
**Middle Orbital Gyrus**	**Left**	**-32**	**56**	**-16**	**24**	**3.28**	**0.001**
**Inferior Parietal Lobule**	**Left**	**-28**	**-44**	**44**	**32**	**3.28**	**0.001**
Postcentral Gyrus	Left	-22	-46	54			0.001
**Superior Parietal Lobule**	**Left**	**-18**	**-56**	**50**	**20**	**3.25**	**0.001**
**White Matter**	**Left**	**-16**	**-54**	**-30**	**40**	**3.2**	**0.001**
**Temporal Pole**	**Right**	**42**	**24**	**-26**	**15**	**3.16**	**0.001**
**Cerebelum IX**	**Right**	**14**	**-44**	**-44**	**91**	**3.11**	**0.001**
White Matter	Right	6	-42	-48			0.001
Cerebelum IX	Left	-6	-44	-44			0.002
Cerebelum IX	Right	2	-50	-46			0.002
**Superior Occipital Gyrus**	**Left**	**-18**	**-86**	**30**	**6**	**3.07**	**0.001**
**Precuneus**	**Left**	**-2**	**-52**	**16**	**10**	**2.99**	**0.001**
**Postcentral Gyrus**	**Right**	**46**	**-26**	**56**	**2**	**2.98**	**0.001**
**Precuneus**	**Left**	**-12**	**-54**	**16**	**1**	**2.98**	**0.001**
**White Matter**	**Left**	**-18**	**-50**	**54**	**6**	**2.98**	**0.001**
**Hippocampus**	**Left**	**-26**	**-6**	**-20**	**13**	**2.95**	**0.002**
**Temporal Pole**	**Right**	**56**	**4**	**-8**	**11**	**2.95**	**0.002**
**White Matter**	**Left**	**-18**	**-40**	**50**	**5**	**2.91**	**0.002**
**IFG p. Orbitalis**	**Right**	**30**	**32**	**-18**	**6**	**2.91**	**0.002**
**Middle Temporal Gyrus**	**Left**	**-44**	**-52**	**16**	**18**	**2.9**	**0.002**
**White Matter**	**Left**	**-2**	**-38**	**22**	**36**	**2.9**	**0.002**
Mid-Cingulate Cortex	Right	6	-42	34			0.003
**Cerebelum IX**	**Left**	**-12**	**-48**	**-36**	**15**	**2.87**	**0.002**
**Precentral Gyrus**	**Right**	**40**	**-18**	**54**	**30**	**2.86**	**0.002**
Precentral Gyrus	Right	46	-18	46			0.002
**Hippocampus**	**Left**	**-32**	**-36**	**0**	**19**	**2.84**	**0.002**
**Cerebelum VIII**	**Left**	**-26**	**-58**	**-42**	**2**	**2.82**	**0.002**
**Precuneus**	**Left**	**-12**	**-56**	**30**	**1**	**2.8**	**0.003**
**Precuneus**	**Left**	**-8**	**-60**	**26**	**2**	**2.8**	**0.003**
**Cerebelum IV-V**	**Right**	**22**	**-46**	**-22**	**4**	**2.8**	**0.003**
**SupraMarginal Gyrus**	**Right**	**52**	**-28**	**36**	**2**	**2.79**	**0.003**
**SupraMarginal Gyrus**	**Right**	**54**	**-36**	**42**	**4**	**2.78**	**0.003**
**Cerebelum VI**	**Right**	**22**	**-52**	**-26**	**4**	**2.77**	**0.003**
Cerebelum X	Right	32	-36	-44	3	2.77	0.003

Location	Hemisphere	X	Y	Z	No. voxels	z-value	Voxel p-value
**Superior Medial Gyrus**	**Left**	**-16**	**58**	**2**	**1**	**2.76**	**0.003**
**White Matter**	**Left**	**-22**	**-40**	**-34**	**4**	**2.76**	**0.003**
**Fusiform Gyrus**	**Right**	**34**	**-30**	**-26**	**2**	**2.75**	**0.003**
**Superior Temporal Gyrus**	**Left**	**-44**	**-42**	**12**	**2**	**2.75**	**0.003**
**Fusiform Gyrus**	**Left**	**-32**	**-40**	**-24**	**4**	**2.74**	**0.003**
**Hippocampus**	**Left**	**-30**	**-16**	**-10**	**5**	**2.74**	**0.003**
**Cerebelum III**	**Right**	**12**	**-32**	**-12**	**3**	**2.73**	**0.003**
**White Matter**	**Right**	**16**	**-14**	**-10**	**1**	**2.72**	**0.003**
**Fusiform Gyrus**	**Left**	**-32**	**-54**	**-12**	**19**	**2.72**	**0.003**
Cerebelum VI	Left	-26	-54	-20			0.004
**Middle Occipital Gyrus**	**Left**	**-36**	**-74**	**34**	**4**	**2.71**	**0.003**
**Hippocampus**	**Right**	**36**	**-36**	**-8**	**1**	**2.7**	**0.003**
**Cerebellar Vermis 6**	**Right**	**0**	**-72**	**-6**	**1**	**2.7**	**0.003**
**Middle Occipital Gyrus**	**Left**	**-40**	**-78**	**20**	**2**	**2.69**	**0.004**
**White Matter**	**Right**	**0**	**12**	**20**	**1**	**2.69**	**0.004**
**Posterior-Medial Frontal**	**Left**	**-6**	**-14**	**54**	**1**	**2.69**	**0.004**
**Mid Orbital Gyrus**	**Left**	**-10**	**42**	**-14**	**3**	**2.68**	**0.004**
**Cerebelum IV-V**	**Right**	**24**	**-28**	**-30**	**1**	**2.68**	**0.004**
**Rectal Gyrus**	**Left**	**-10**	**34**	**-18**	**2**	**2.67**	**0.004**
**Temporal Pole**	**Left**	**-40**	**26**	**-26**	**1**	**2.67**	**0.004**
**Postcentral Gyrus**	**Left**	**-36**	**-36**	**50**	**1**	**2.66**	**0.004**
**Precentral Gyrus**	**Right**	**34**	**-12**	**56**	**1**	**2.65**	**0.004**
**Posterior Cingulate Cortex**	**Left**	**-6**	**-52**	**22**	**2**	**2.65**	**0.004**
**Hippocampus**	**Left**	**-26**	**-10**	**-12**	**1**	**2.65**	**0.004**
**ParaHippocampal Gyrus**	**Left**	**-24**	**-32**	**-16**	**1**	**2.64**	**0.004**
**Rolandic Operculum**	**Left**	**-44**	**-4**	**2**	**6**	**2.64**	**0.004**
**Superior Temporal Gyrus**	**Right**	**46**	**-24**	**2**	**4**	**2.63**	**0.004**
**Middle Occipital Gyrus**	**Left**	**-36**	**-70**	**32**	**2**	**2.63**	**0.004**
**Fusiform Gyrus**	**Left**	**-22**	**-32**	**-24**	**2**	**2.63**	**0.004**
**White Matter**	**Right**	**16**	**-40**	**-36**	**1**	**2.63**	**0.004**
**Middle Occipital Gyrus**	**Left**	**-32**	**-66**	**28**	**1**	**2.63**	**0.004**
**Fusiform Gyrus**	**Left**	**-32**	**-60**	**-12**	**1**	**2.62**	**0.004**
**Cerebelum IV-V**	**Right**	**18**	**-46**	**-18**	**3**	**2.62**	**0.004**
**Postcentral Gyrus**	**Left**	**-40**	**-32**	**58**	**1**	**2.61**	**0.004**
**Rectal Gyrus**	**Left**	**-6**	**54**	**-22**	**3**	**2.61**	**0.005**
**Cerebelum Crus 1**	**Right**	**10**	**-92**	**-26**	**1**	**2.61**	**0.005**
**Inferior Temporal Gyrus**	**Left**	**-42**	**-46**	**-8**	**1**	**2.6**	**0.005**
**Cerebelum III**	**Right**	**16**	**-28**	**-24**	**1**	**2.6**	**0.005**
**Pallidum**	**Left**	**-20**	**-8**	**-4**	**1**	**2.59**	**0.005**
**White Matter**	**Right**	**24**	**-54**	**-38**	**1**	**2.59**	**0.005**
**White Matter**	**Right**	**20**	**-44**	**-42**	**2**	**2.59**	**0.005**
**Hippocampus**	**Left**	**-34**	**-10**	**-16**	**1**	**2.59**	**0.005**
**White Matter**	**Left**	**-14**	**-34**	**56**	**1**	**2.59**	**0.005**
**White Matter**	**Left**	**-8**	**-24**	**-24**	**1**	**2.58**	**0.005**

SI References

1. Taylor JSH, Davis MH, Rastle K. Comparing and validating methods of reading instruction using behavioural and neural findings in an artificial orthography. J Exp Psychol Gen. 2017;146(6):826-58.
2. Friston K, Ashburner J, Frith C, B. PJ, Heather J, Frackowiak R. Spatial registration and normalization of images. Human Brain Mapping. 1995;2:165–89.
3. Ashburner J, Friston KJ. Multimodal image coregistration and partitioning – A unified framework. NeuroImage. 1997;6:209–17.
4. Ashburner J, Friston KJ. Unified segmentation. NeuroImage. 2005;26:839–51.
5. Josephs O, Henson R. Event-related functional magnetic resonance imaging: Modelling, inference and optimization. Philosophical Transactions of the Royal Society B: Biological Sciences. 1999;354(1387):1215-28.
6. Whitney C. How the brain encodes the order of letters in a printed word: the SERIOL model and selective literature review. Psychon Bull Rev. 2001;8(2):221-43.
7. Tzourio-Mazoyer N, Landeau B, Papathanassiou D, Crivello F, Etard O, Delcroix N, et al. Automated anatomical labeling of activations in SPM using a macroscopic anatomical parcellation of the MNI MRI single-subject brain. NeuroImage. 2002;15(1):273-89.
